# Chemical profiling of selected Ayurveda formulations recommended for COVID-19

**DOI:** 10.1186/s43088-020-00089-1

**Published:** 2021-01-11

**Authors:** Sulaiman C. T., Deepak M., Ramesh P. R., Mahesh K., Anandan E. M., Indira Balachandran

**Affiliations:** 1 Phytochemistry Division, Centre for Medicinal Plants Research, Arya Vaidya Sala, Kottakkal, Malappuram, Kerala 676503 India; 2 Clinical Research Department, Arya Vaidya Sala, Kottakkal, Kerala India; 3 Product Development Department, Arya Vaidya Sala, Kottakkal, Kerala India

**Keywords:** COVID-19, Ayurveda, Indukantham Kwatham, Vilvadi Gulika, Mukkamukkatuvadi Gulika, HPTLC

## Abstract

**Background:**

The novel coronavirus disease 2019 (COVID-19), caused by the severe acute respiratory syndrome coronavirus-2 (SARS-CoV-2), is the global health concern since December 2019. It has become a big challenge for the researchers to find a solution for this newly evolved pandemic. In Ayurveda point of view, COVID-19 is a *Janapadodhwamsa vikara* (epidemic disease), a situation where the environment—air, water, land, and seasons—is vitiated, causing a simultaneous manifestation of a disease among large populations. The aim of this study is to identify the active compounds of selected Ayurveda medicines recommended for COVID-19.

**Results:**

The selected preparations are traditionally recommended for the management of various kinds of fever including the infectious ones and to enhance the immunity. HPTLC analysis of the same showed presence of many active molecules like umbelliferone, scopoletin, caffeic acid, ferulic acid, gallic acid, piperine, curcumin, berberine, and palmatine.

**Conclusion:**

The study provided valuable scientific data regarding the active ingredients of the selected medicines with proven therapeutic potentials like anti-viral, immunomodulatory, and anti-inflammatory activities.

## Background

COVID-19 has emerged as the most dangerous global pandemic threat since its outbreak during December 2019 in Wuhan, China. As of September 4, 2020, the World Health Organization (WHO) has reported more than 26 million confirmed cases and 8.6 lakhs deaths worldwide and it has spread to 216 countries, areas, or territories (https://www.who.int/emergencies/diseases/novel-coronavirus-2019). Now, it is a big challenge for the researchers and health professionals to find out a solution for this deadly viral infection. COVID-19 is a viral infection that has been known to have the fastest frequency of replication in its positive strand resulting in the quick development of new progeny viral cells inside the host cells. SARS-CoV-2 is a single-stranded RNA pathogen, which is characterized by a high mutation rate [1, 2]. In Ayurveda point of view, COVID-19 is a *Janapadodhwamsa vikara* (epidemic disease), a situation where the environment—air, water, land, and seasons—is vitiated, causing a simultaneous manifestation of a disease among large populations [3].

Medicinal plants have been used as a treatment and defensive strategy for several infectious diseases since ancient times. The benefit of using these herbs in viral respiratory infections is to build immune-stimulating and inflammation-modulating effects to prevent severe life-threatening conditions. Holistic approach of Ayurveda focuses on prevention of diseases through lifestyle modification, dietary management, prophylactic interventions for improving the immunity, and managing the symptoms using herbal preparations. Medicinal plants have been reported to have anti-viral activity and many species such as *Aegle marmelos*, *Andrographis paniculata*, *Acacia nilotica*, *Ocimum tenuiflorum*, *Piper nigrum*, *Solanum nigrum*, and *Terminalia chebula* have been scientifically proved for their anti-viral properties [4–6]. Ayurveda medicines were recommended by the Ministry of AYUSH, Government of India to enhance the immunity and to prevent the severe conditions of Cov-2 infection. Detailed guidelines have been published by the AYUSH Ministry regarding the management of COVID-19 (https://www.ayush.gov.in/ayush-guidelines.html). About 80% of COVID-19 cases are with mild symptoms requiring only primary medical care. Ayurveda medicines are advised to patients with mild symptoms and those under surveillance which addresses the therapeutic province within an integrative model of care [7]. The present study was focused on the identification of active ingredients of certain Ayurveda medicines such as Indukantham Kwatham (IK), Vilvadi Gulika (VG), and Mukkamukkatuvadi Gulika (MMG) in which the ingredient plants have been reported to possess immunomodulatory and anti-viral properties.

*Indukantham Kwatham* is a polyherbal tablet prepared out of specific parts of different medicinal plants such as *Holoptelea integrifolia*, *Cedrus deodara*, *Gmelina arborea*, *Aegle marmelos*, *Stereospermum colais*, *Oroxylum indicum*, *Premna corymbosa*, *Desmodium gangeticum*, *Pseudarthria viscida*, *Solanum anguivi*, *Solanum virginianum*, *Tribulus terrestris*, *Piper longum*, *Piper mullesua*, *Plumbago zeylanica*, and *Zingiber officinale*. It is generally used for the treatment of intermittent fever and fatigue and to enhance the resistance power [8]. *Vilvadi Gulika* is prepared using different parts of the various medicinal plants such as *Aegle marmelos*, *Ocimum tenuiiflorum*, *Pongamia pinnata*, *Veleriana jatamansi*, *Cedrus deodara*, *Terminalia chebula*, *Phyllanthus emblica*, *Terminalia bellirica, Zingiber officinale*, *Piper nigrum*, *Piper longum*, *Curcuma longa*, and *Berberis aristata*. The ingredient plants of *Mukkamukkatuvadi Gulika* are *Terminalia chebula*, *Phyllanthus emblica*, *Terminalia bellirica*, *Zingiber officinale*, *Piper nigrum*, *Piper longum*, *Cuminum cyminum*, *Nigella sativa*, *Acorus calamus*, *Swertia chirata*, *Cinnamomum camphora*, *Myristica fragrans*, *Aloe vera*, *Syzygium aromaticum*, *Allium sativum*, *Piper cu*beba, *Saussurea costus*, *Cinnamomum verum*, *Ferula assa-foetida*, *Trchyspermum roxburghianum*, and *Vitex negundo* [8].

## Methods

### Chemicals and reagents

Chemicals such as toluene (CN: IE5F650118), ethyl acetate (CN: ID5F650128), and methanol (CN: SA5P650021) were procured from Merck India.

### Sample preparation

The selected medicines were obtained from the Product Development Department of the Institute. Two grams each of IK (Batch No. 198339), VG (Batch No. 193083), and MMG (Batch No. 194967) were sonicated with chromatographic grade methanol in an ultra-sound bath (Labnet Scientific, India) for 20 min. It was then filtered through a membrane filter (0.45 μm) and kept under the refrigerator until HPTLC analysis.

### Instruments and general chromatographic conditions

HPTLC analysis was carried out by the CAMAG HPTLC system (Switzerland). Samples were applied using CAMAG ATS 4 auto sampler on aluminum backed pre-coated silica gel 60F_254_ HPTLC plate (Merck India). Mobile phase was optimized as toluene, ethyl acetate, and methanol in the ratio of 7:3:1. The chromatogram was developed in a saturated Twin Trough chromatographic chamber (Camag, Switzerland) and was visualized under UV-chamber (254 and 366 nm) and in visible light after derivatizing with anisaldehyde sulfuric acid reagent followed by heating at 105 °C for 5 min.

## Results

**Fig. 1:**
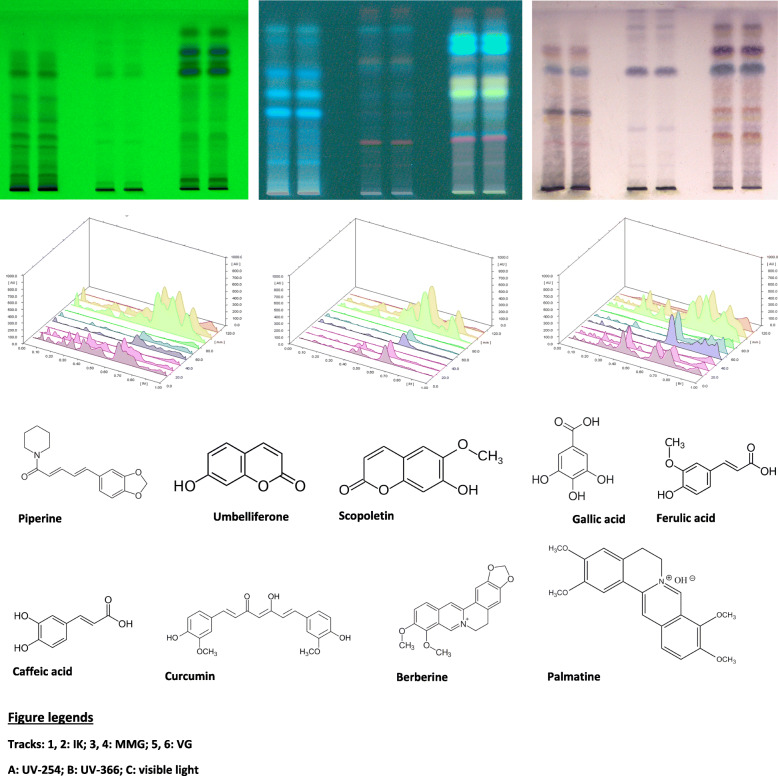
HPTLC profiling of IK, MMG, and VG documented at UV-254, 366, and visible light

Rapid chromatographic method has been developed for the chemical fingerprinting of selected medicines by modern high-performance thin-layer chromatography. The optimized mobile phase provided good resolution under various documentation systems such as UV-254, 366, and visible light. Chromatogram and 3D-illustrated display are presented in Fig. 1. HPTLC analysis showed presence of various compounds belonging to different groups of phytochemicals such as alkaloids, coumarins, and phenolics. Structural identification was confirmed with matching *R*_f_ of standard compounds. The compounds identified from the tested formulations are given in Table 1.

**Table 1:** Compounds identified from IK, MMG, and VG by HPTLC analysis

Sl. no.	Compounds	*R*_f_ value	Present in
1	Caffeic acid	0.21	IK, VG
2	Palmatine	0.33	VG
3	Ferulic acid	0.37	IK, VG
4	Gallic acid	0.43	IK, VG, MMG
5	Scopoletin	0.44	IK, VG, MMG
6	Berberine	0.45	VG
7	Umbelliferone	0.57	IK, VG, MMG
8	Piperine	0.71	MMG
9	Curcumin	0.73	VG

Coumarins such as umbelliferone and scopoletin are detected in all the three selected medicines and these are the plant coumarins reported from many medicinal plants. Phenolic compounds like caffeic acid and ferulic acid are found in both IK and VG. Gallic acid was identified from all the three selected medicines and has been reported from many ingredient plants. VG showed the presence of alkaloids such as curcumin, berberine, and palmatine which might have extracted from its ingredient plants like *Piper nigrum*, *Piper longum*, *Curcuma longa*, and *Berberis aristata*.

## Discussion

Bioactive compounds from natural products are attractive candidates for drug development. Numerous medicinal plants have been reported to possess various therapeutic properties including anti-viral, anti-inflammatory, and immunomodulatory activities. The chemical profiling of three selected medicines showed the presence of various biologically active compounds belonging to different classes of phytochemicals such as alkaloids, phenolics, and coumarins. Coumarins such as umbelliferone and scopoletin were found to be common for all the selected samples. Naturally occurring coumarins have been reported to possess diverse biological and pharmacological properties such as anti-viral, anti-coagulant, anti-bacterial, anti-fungal, anti-protozoal, insecticidal, fungicide, anti-mycobacterial, anti-mutagenic, anti-amnesic, and anti-inflammatory activities [9, 10]. There are numerous evidences for the inhibitory role of coumarins against infection of various viruses such as HIV, influenza, enterovirus 71, and coxsackievirus A16. The mechanisms involve either inhibition of proteins essential for viral entry, replication, and infection or regulation of cellular pathways such as Akt-Mtor, NF-κB, and anti-oxidative pathways including NrF-2 [11].

Alkaloids such as piperine, curcumin, berberine, and palmatine were identified from the selected medicines. Piperine is detected from both IK and MMG and that might have come from the ingredient plant *Piper nigrum*. The immunomodulatory potential of piperine has been reported earlier [12]. Piperine was reported to inhibit proliferative response induced by lipopolysaccharide (LPS) and immunoglobulin α-IgM antibody and resulted in inhibition of IgM antibody secretion and reduced expression of cluster of differentiation CD86 [13]. Another study by Lee et al. 2018 [14] demonstrated that piperine in combination with gamma-aminobutyric acid (GABA) mediated p38 and JNK MAPK activation, which increased EPO and EPO-R expression, resulting in upregulation of IL-10 and NF-κB. Alkaloids like curcumin, berberine, and palmatine were identified from VG. The anti-viral effect of curcumin on Zika and chikungunya viruses has been well established [15]. The literature showed that curcumin mediates its anti-viral activity through various mechanisms. Curcumin has been reported to inhibit the Japanese encephalitis virus by dysregulated ubiquitin-proteasome system and an accumulation of ubiquitinated proteins [16]. Curcumin was also reported to inhibit various virus replications like Rift Valley fever virus and hepatitis C virus [17, 18]. Moreover, curcumin was shown to impact HCV replication through binding and fusion [19] and similar results were reported in the case of ZIKV and CHIKV. HIV-1 integrase activity of curcumin was also reported previously [20]. Various pharmacological activities of berberine such as anti-oxidant, anti-bacterial, anti-inflammatory, anti-viral, nephroprotective, and cardioprotective have been reported earlier [21]. Anti-viral activity of berberine against human cytomegalovirus has been reported previously [22]. The immunomodulatory effect of berberine was validated in many previous literatures [23–25].

Currently, there are no available vaccines or specific medicines for the treatment of COVID-19. In light of the outbreak, various treatment modalities have been considered, including herbal medicine, which has been widely used during the past epidemic outbreaks, such as severe acute respiratory syndrome (SARS) and H1N1 influenza. The phytochemicals identified from IK, MMG, and VG are active molecules with potential biological properties such as anti-viral, anti-inflammatory, and immunomodulatory activities.

## Conclusion

COVID-19 pandemic is a global challenge for human health, and researchers are urgently seeking medicine for it. Currently, the treatment options for COVID-19 are limited due to non-availability of vaccines or specific medicines. In this context, the search for traditional herbal medicine is also a viable strategy for COVID-19 management. The present study on selected Ayurveda medicines provided valuable scientific data regarding the active ingredients of the drugs tested with proven therapeutic potentials like anti-viral, immunomodulatory, and anti-inflammatory activities.

## Data Availability

The datasets used and/or analyzed during the current study are available from the corresponding author on reasonable request.

## Abbreviations

IK: Indukantham Kwatham
VG: Vilvadi Gulika
MMG: Mukkamukkatuvadi Gulika
HPTLC: High-performance thin-layer chromatography
